# Single-cell analysis of somatic mutation burden in mammary epithelial cells of pathogenic *BRCA1/2* mutation carriers

**DOI:** 10.1172/JCI148113

**Published:** 2022-03-01

**Authors:** Shixiang Sun, Kristina Brazhnik, Moonsook Lee, Alexander Y. Maslov, Yi Zhang, Zhenqiu Huang, Susan Klugman, Ben H. Park, Jan Vijg, Cristina Montagna

**Affiliations:** 1 Department of Genetics, Albert Einstein College of Medicine, New York, New York, USA.; 2 Laboratory of Applied Genomic Technologies, Voronezh State University of Engineering Technologies, Voronezh, Russia.; 3 Department of Radiation Oncology, Rutgers Cancer Institute of New Jersey, New Brunswick, New Jersey, USA.; 4 Division of Reproductive and Medical Genetics, Department of Obstetrics & Gynecology and Women’s Health, Montefiore Medical Center, Albert Einstein College of Medicine, New York, New York, USA.; 5 Vanderbilt-Ingram Cancer Center, Vanderbilt University Medical Center, Nashville, Tennessee, USA.; 6 Center for Single-Cell Omics, School of Public Health, Shanghai Jiao Tong University School of Medicine, Shanghai, China.

**Keywords:** Genetics, Breast cancer, Genetic instability

## Abstract

Inherited germline mutations in the breast cancer gene 1 (*BRCA1*) or *BRCA2* genes (herein *BRCA1/2*) greatly increase the risk of breast and ovarian cancer, presumably by elevating somatic mutational errors as a consequence of deficient DNA repair. However, this has never been directly demonstrated by a comprehensive analysis of the somatic mutational landscape of primary, noncancer, mammary epithelial cells of women diagnosed with pathogenic *BRCA1*/*2* germline mutations. Here, we used an accurate, single-cell whole-genome sequencing approach to first show that telomerized primary mammary epithelial cells heterozygous for a highly penetrant *BRCA1* variant displayed a robustly elevated mutation frequency as compared with their isogenic control cells. We then demonstrated a small but statistically significant increase in mutation frequency in mammary epithelial cells isolated from the breast of *BRCA1/2* mutation carriers as compared with those obtained from age-matched controls with no genetically increased risk for breast cancer.

## Introduction

Breast cancer is the most common cancer in women worldwide (1). Up to 10% of breast cancer is due to genetic predisposition (2), with inherited mutations in breast cancer gene 1 (*BRCA1*) or *BRCA2* (herein referred to as *BRCA1/2*) accounting for most cases. For other germline risk variants (e.g., ATM serine/threonine kinase [*ATM*], partner and localizer of BRCA2 [*PALB2*], and checkpoint kinase 2 [*CHEK2*]), susceptibility to breast cancer has been estimated to account for less than 50% of cases. This percentage is significantly higher than the lifetime risk for sporadic breast cancer, which accounts for no more than 15% of cases (3). *BRCA1/2* and other known hereditary cancer genes are involved in DNA repair, and defects in their functions likely underlie increased spontaneous mutation frequency. Because cancer is caused by DNA mutations, a higher mutation rate in mammary epithelial cells could explain the increased risk for breast cancer in women who carry such genetic defects, as well as the higher risks for developing cancer at other anatomical sites (4). While conceivable, this has never been demonstrated in vivo, and the frequency and type of mutations affecting human mammary epithelial cells (HMECs) of *BRCA1/2* carriers who are women before tumor development remain unknown.

Somatic mutations in primary human cells or tissues are remarkably difficult to analyze, as they are randomly distributed across the genome, are of very low abundance, and are mostly unique to each cell (5). To overcome these challenges, we developed previously a single-cell whole-genome sequencing method to detect mutations in individual cells isolated from primary tissues using bulk genome sequencing to correct for germline variants (6). This method, which uncovered age-related somatic mutational landscapes in primary human lymphocytes and hepatocytes (7, 8), was applied here to measure somatic mutation burden in noncancer primary mammary epithelial cells of *BRCA1/2* germline mutation carriers and controls.

## Results and Discussion

**Figure 1:**
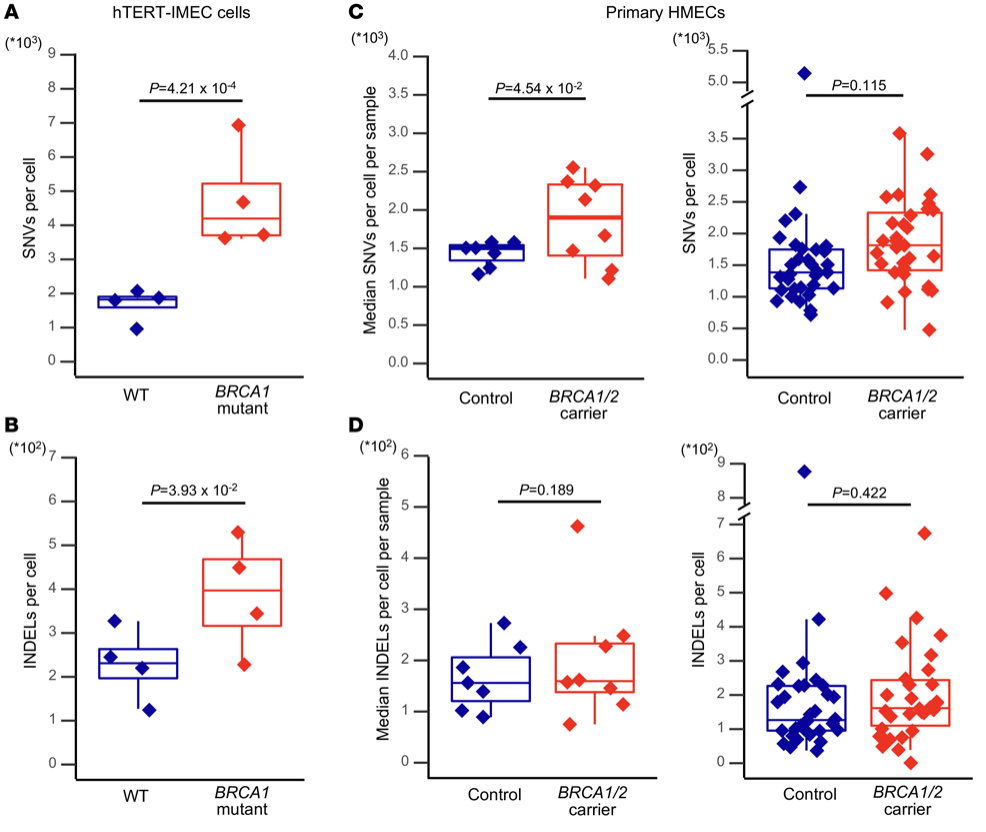
Mutation levels in human mammary epithelial cells. (**A**) SNV and (**B**) INDEL levels in hTERT-IMEC WT (blue) and *BRCA1* mutant (red) cells (*n =* 4 for each type). (**C**) SNV and (**D**) INDEL levels in primary HMECs. Graphs on the left in **C** and **D** depict the median mutations per sample in both groups (control: blue, *n =* 7; carrier: red, *n =* 8; negative binomial generalized linear model), while graphs on the right depict the distributions of single HMECs in control (blue, *n =* 32) and *BRCA1/2* mutant carrier (red, *n =* 31) groups (negative binomial generalized linear mixed-effect model).

To verify that *BRCA1* haploinsufficiency increases the frequency of single-nucleotide variants (SNVs) and small insertions and deletions (INDELs) in mammary epithelial cells, we first compared a strain of primary telomerized mammary epithelial cells (human telomerase reverse transcriptase–immortalized mammary epithelial cells [hTERT-IMECs]) heterozygous for 185delAG, a pathogenic and highly penetrant *BRCA1* mutation, to isogenic WT control hTERT-IMECs (9). Two isogenic *BRCA1* mutant clones, het #1 and het #2, 2 cells each, were examined in comparison to their WT control cells. Analysis of 4 individual cells per genotype (Supplemental Table 1; supplemental material available online with this article; https://doi.org/10.1172/JCI148113DS1) indicated a significant increase of both SNVs and INDELs (2.3-fold increase in SNVs and 1.7-fold increase in INDELs) in hTERT-IMEC *BRCA1* heterozygous mutant cells as compared with isogenic WT cells (i.e., for mutant and control cells, respectively, 4196 ± 1536 SNVs and 1825 ± 473 SNVs per cell and 397 ± 129 INDELs and 231 ± 82 INDELs per cell; *P* = 4.21 × 10^-4^ and *P* = 3.93 × 10^-2^, respectively; negative binomial generalized linear model [NBGLM]) (Figure 1, A and B, and Supplemental Table 2).

To establish in vivo relevance of these results, we isolated nontumor mammary epithelial cells from women diagnosed with a *BRCA1/2* germline mutation (8 individuals for a total of 31 cells; Supplemental Figure 1 and Supplemental Table 1) as well as from age-matched women undergoing reduction mammoplasty purely for cosmetic reasons used as controls (7 individuals for a total of 33 cells). From each individual 2–8 single primary HMECs were sequenced alongside genomic DNA obtained from bulk mammary gland tissue of the same individuals to correct for germline variants.

First, we calculated the median SNV frequency of all cells for each individual and then compared the *BRCA1/2* mutant and control groups. The median SNV frequency per individual was significantly elevated (1.3-fold) in the *BRCA1/2* germline mutation carrier group as compared with the control group (*P* = 4.54 × 10^-2^, NBGLM) (i.e., 1902 ± 561 SNVs and 1506 ± 163 SNVs, respectively, excluding 1 outlier cell, M10-1, with 5143 SNVs in a control, as identified by Tukey’s test) (Figure 1C, left; Supplemental Figure 2, A and B; and Supplemental Table 2). In addition, we found an outlier cell with high SNV frequency in human B lymphocytes (7). We compared the average number of SNVs per cell across all cells between the 2 groups. This essentially confirmed the elevated mutation frequency in *BRCA1/2* mutation carriers, albeit this was not statistically significant (*P* = 0.115; negative binomial generalized linear mixed-effect model [NBGLMM]), with 1814 ± 682 SNVs and 1383 ± 455 SNVs per cell on average, for mutant and control cells, respectively, excluding the same outlier cell (Figure 1C, right). In contrast to the increase in hTERT-IMEC *BRCA1* heterozygous mutant cells, there were no statistically significant differences in the frequencies of INDELs between the mutant and control groups in either median INDEL frequency per individual (i.e., 160 ± 120 INDELs and 156 ± 66 INDELs, respectively) or the average number per cell (i.e., 162 ± 143 INDELs and 126 ± 155 INDELs, respectively) (*P* = 0.189 and *P* = 0.422, NBGLM and NBGLMM, respectively; Figure 1D and Supplemental Figure 2C).

These results indicate a more modest effect of heritable pathogenic *BRCA1/2* germline mutations on somatic mutations in primary cells in vivo compared with established isogenic hTERT-IMEC strains in vitro. Of note, both the control and *BRCA1*-defective cells of the in-culture model had higher mutation frequencies than the primary cells obtained in vivo from *BRCA1/2* controls and women with the *BRCA1/2* mutation, likely as a consequence of replication errors accumulated during extensive passaging of the hTERT-IMEC strains. Furthermore, this effect was stronger in the *BRCA1*-defective cells than in the control cells, presumably due to the extended replication in a DNA double-strand break (DSB) repair–defective background of the clonally derived *BRCA1* mutants.

Interestingly, among the *BRCA1*-deficient hTERT-IMECs, 2 subgroups differing in their mutation frequencies could be identified (Figure 1A), with cells from clone het #2 showing more SNVs and INDELs than cells from clone het #1. We found that the relatively high mutation frequency in het #2 cells was associated with a deleterious missense mutation in tumor protein P53 (*TP53*; Supplemental Table 3). Cells from this clone were also reported to have reduced survival after treatment with γ radiation (9). These findings suggest a relatively low capacity of het #2 cells to cope with DNA damage, possibly resulting in more mutations relative to het #1 cells. Of note, a slight increase in mutation frequency has been previously reported in p53-defective mice (10).

In primary HMECs, cells from 4 individuals (M05, M08, M21 and M27) were found to display higher SNV frequencies than others in the *BRCA1/2* mutant group (Supplemental Figure 2A). We found no deleterious somatic mutations (CADD score, ≥15) in 518 genes previously established to be involved in genome maintenance in these cells (ref. 11 and Supplemental Table 3). To evaluate if any germline mutations other than *BRCA1/2* could be a cause for the overall higher mutation frequency of cells in the 4 individuals, we analyzed their bulk DNA. While we confirmed the *BRCA1/2* mutations in these individuals, no other pathogenic germline mutations in any of the 518 genome maintenance genes were shared by these 4 individuals. However, individual M21 presented a higher INDEL frequency than any other *BRCA1/2* mutant carrier (Supplemental Figure 2C) and contained a pathogenic germline copy number variation in *ERCC2* (ERCC excision repair 2, TFIIH core complex helicase subunit), which is involved in nucleotide excision repair.

We also excluded the presence of pathogenic germline *BRCA1/2* mutations in the control group using bulk sequencing data (Supplemental Table 3). Individual M10, who had the high-SNV frequency outlier cell M10-1 (Supplemental Table 3), was found to have a pathogenic germline copy number variation in *PALB2*, a pivotal player in DNA DSB repair, which could suggest a weak capability of M10 in coping with DNA damage (12). In addition, the SNV outlier cell M10-1 carried deleterious somatic mutations in fragile histidine triad diadenosine triphosphatase (*FHIT*) and tripartite motif containing 67 (*TRIM67*) (Supplemental Table 4); both are associated with the DNA damage response (13, 14), indicating unique DNA repair deficiencies of outlier cell M10-1. Another individual, M25, in the control group presented a high-INDEL frequency outlier cell, M25-1, but without pathogenic germline mutations found in genome maintenance genes. Furthermore, we found no deleterious somatic mutations in DNA damage response genes for outlier cell M25-1.

**Figure 2:**
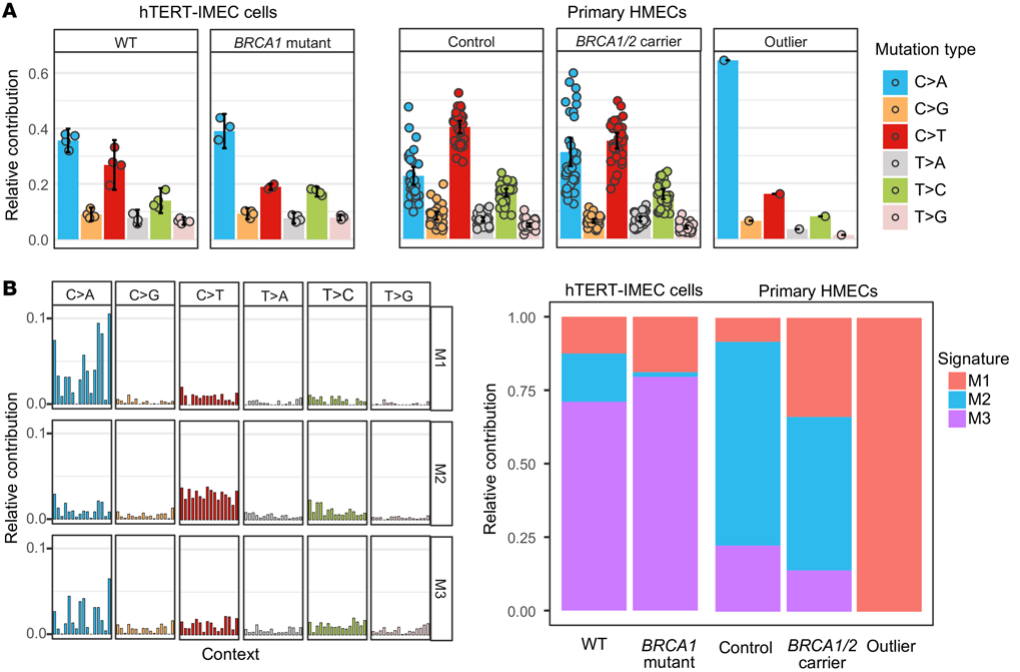
Mutational spectra in human mammary epithelial cells. (**A**) Relative contribution of 6 mutation types to the point mutation spectrum for the indicated mammary sample groups. Data are shown as the mean and 95% confidence intervals of the relative contribution of each mutation type in hTERT-IMEC WT (*n =* 4) and *BRCA1* mutant (*n =* 4) sample groups and HMEC control sample groups (31 cells from 7 participants), *BRCA1/2* mutant carrier sample groups (31 cells from 8 participants), and the outlier cell from the control group. (**B**) Three mutational signatures (M1, M2, and M3) were de novo identified by nonnegative matrix factorization analysis of the somatic mutations in the different groups in **A**. “Context” on the *x* axis represents the mutational profile using the conventional 96 mutation–type classification in COSMIC. This classification is based on the 6 substitution subtypes shown on top, as well as the nucleotides immediately 5′ and 3′ to the mutation (the sorting order is A, C, G and T). The contributions of M1, M2, and M3 signatures to all SNVs in these 5 groups using the nonnegative matrix factorization method is shown on the right.

Next, we analyzed the mutation spectra to explore the possible source of detected somatic mutations in mammary epithelial cells. We first analyzed the SNVs in the hTERT-IMEC WT and *BRCA1* mutant cells, the primary HMECs obtained from the *BRCA1/2* carrier group, as compared with controls, and the outlier cell separately (Figure 2A and Supplemental Figure 3A). Using nonnegative matrix factorization (NMF) we extracted 3 de novo mutational signatures (signatures M1, M2, and M3) from the mutation spectra of these 5 groups (Figure 2B). The extracted signatures were confirmed by analysis using hierarchical Dirichlet process (Supplemental Figure 3, B and C; Supplemental Table 5; and Supplemental Methods). The results using NMF showed significantly different contributions of mutation signatures between *BRCA1/2* mutant carriers and controls with signature M1 as the major contributor to the differences between the 2 groups (*P* < 2.2 × 10^-16^, Pearson’s χ^2^ test; Supplemental Table 5). Signature M1, dominated by GC to TA transversions at non-CpG sites, was significantly enriched in *BRCA1/2* mutant samples (mutant [95% confidence interval] as compared with WT controls [95% confidence interval], 0.179 [0.156–0.204] vs. 0.114 [0.072–0.156] in hTERT-IMECs and 0.297 [0.278–0.318] vs. 0.069 [0.050–0.089] in HMECs; Supplemental Figure 3D). M1 also dominated the outlier cell(0.999 [0.990–1.000]). M1 is highly similar to COSMIC signatures associated with reactive oxygen species (cosine similarity: 0.895 [SBS18; associated with defective base excision repair] and 0.893 [SBS36; similar to SBS18 in aetiology]; Supplemental Table 5 and refs. 15, 16). It is possible that signature M1 relates to the reported role of *BRCA1/2* in protection against reactive oxygen species through base excision repair (17). Importantly, the higher contributions of M1 in the *BRCA1/2* groups point to the underlying tumor risk in mutation carriers, as it is similar to breast cancer–related signature SBS18 (18, 19).

Contrary to M1, M2 was predominant in the control group (mutant vs. WT controls, 0.019 [0.001–0.042] vs. 0.187 [0.141–0.235] in hTERT-IMECs and 0.571 [0.549–0.593] vs. 0.728 [0.705–0.754] in HMECs; Supplemental Figure 3D). M2 is similar to the clock-like signature SBS5 (cosine similarity: 0.861), while the enrichment of SBS5 in the HMEC control group was retained when we decomposed the SNV patterns using known signatures from COSMIC (Supplemental Figure 3E). Finally, another GC to TA transversion occurring mainly on CpT and CpA positions dominated signature M3, enriched in the hTERT-IMECs (mutant vs. WT controls, 0.802 [0.770–0.830] vs. 0.699 [0.640–0.760]) and was minor in HMECs (mutant vs. WT controls, 0.132 [0.106–0.158] vs. 0.202 [0.173–0.229]). This suggests a different type of damage induced by culture conditions, namely, oxidative damage acquired during culture conditions under ambient oxygen levels (20), which was also reported for clonal organoid cultures (cosine similarity: 0.861; ref. 21).

Next, we analyzed the INDEL spectra of the hTERT-IMEC lines and the primary HMECs using NMF methods (Supplemental Figure 4A) and extracted 2 de novo mutational signatures (IDM1 and IDM2; Supplemental Figure 4B). IDM1, characterized predominantly by insertions at ≥5 bp mononucleotide thymine repeats, represented the mutation spectrum of primary HMECs and is highly similar to clock-like signature ID1 (cosine similarity: 0.832; Supplemental Table 5). IDM2, enriched with insertions at ≥5 bp and deletions at ≥6 bp mononucleotide thymine repeats, dominated the hTERT-IMECs (Supplemental Figure 4B). This difference between INDEL signatures in hTERT-IMEC lines and primary HMECs confirms the observations on SNVs, suggesting that different types of mutations accumulate in culture and in vivo. No differences between *BRCA1/2* mutant and control groups were found (Supplemental Figure 4C), not even after decomposing the INDEL patterns using known signatures from COSMIC (Supplemental Figure 4D).

To test if the observed somatic variants could have emerged from clonal expansion of ancestral stem cells, we analyzed the different single cells in each individual for shared mutations (Supplemental Figure 5 and Supplemental Table 6). We found very few somatic mutations shared between single cells of primary HMECs. The highest number was found between 2 of M31’s cells, M31-1 and M31-3, with 3% of mutations shared by only these 2 cells (Supplemental Table 6). No overlap was found in 4 individuals, while in the other individuals, the number of shared SNVs was small (Supplemental Table 6), indicating very recent occurrence. In HMECs, clonal expansion analysis was insufficiently powered, as expected, with only a few single cells from each individual expected to hit few major clones.

In the clonally derived hTERT-IMECs, one would expect to find more extensive overlap of mutations, even with only a few cells analyzed, and this is indeed what we found (Supplemental Figure 5A). In the control cells, about 5% of all mutations were shared, mostly between cells IMEC-wt3 and IMEC-wt4. In the *BRCA1* heterozygous mutant cells, the percentage of overlapping mutations was much higher (i.e., 31% in het #1 and 17% in het #2), undoubtedly as consequence of these cells being derived from knockin clones (9). The numbers were high enough to generate a phylogenetic tree that indicates the history of evolution of somatic mutations in these cells (Supplemental Figure 5B).

In summary, using advanced single-cell sequencing methods, we characterized for the first time to our knowledge the landscape of somatic mutations in normal mammary epithelial cells in vivo in women diagnosed with a *BRCA1*/*2* heterozygous germline mutation. The results indicated robustly increased SNV and INDEL frequencies in the *BRCA1* knockin clones in vitro as compared with their isogenic controls. SNV frequencies were also significantly higher in *BRCA1/2* carriers in which mammary epithelial cells were directly isolated from primary human tissues, albeit the difference was much smaller. The more robust *BRCA1* effect on mutation frequency in culture was likely due to exposure to oxygen during extended passaging of the isogenic hTERT-IMEC *BRCA1* mutant cells, which would give rise to replication errors in a haploinsufficient DSB repair background. However, we cannot rule out the possibility of highly mutated HMECs in vivo being not viable or giving rise to neoantigens and being eliminated through the immune system or other surveillance mechanisms (22). Mutation signature analysis suggests that the most likely source of the mutations in the *BRCA1/2* mutant carriers is oxygen-free radicals.

*BRCA1* deficiency should give rise to less efficient DNA DSB repair by homologous recombination (HR) as a consequence of the gene-dose effect. While HR is considered to be an error-free repair process, it can be highly mutagenic because of the DNA synthesis steps in various stages of the process (23). It is possible that reduced HR capacity would increase DNA synthesis errors, but the possibility that alternative pathways are involved should also be considered. As we inferred from mutation signature analysis, it is possible that *BRCA1/2* is involved in repair pathway(s) for small-base damage and DNA single-strand breaks, with impaired *BRCA1/2* function contributing to somatic SNVs, with patterns associated with pretumor development of breast cancer.

Finally, the dramatic increase in sequencing-based technology used to assess somatic mutations at the single-cell level in vivo (24) can be expected to lead to increased precision in cancer diagnostic approaches. Our current findings may have important clinical implications, such as aiding stratification of tumor risk by evaluating SNV levels or the accumulation of mutation spectra (e.g., SBS18) in conjunction with predicted pathogenicity scores (25, 26).

## Methods

Details regarding the experimental methods and statistical analyses are included in the Supplemental Methods.

### Data and materials availability.

Whole-genome sequencing data (dbGaP accession phs002411.v1.p1) can be accessed at https://www.ncbi.nlm.nih.gov/projects/gap/cgi-bin/study.cgi?study_id=phs002411.v1.p1

### Study approval.

Informed consent was obtained from all individuals who contributed biological specimens to the study. Experimental procedures were approved by the Internal Review Boards of the Albert Einstein College of Medicine (IRB 13-2012, subprotocol to IRB 2013-2012).

## Author contributions

JV and CM conceived of and supervised the study. KB, ML, YZ, and ZH performed the experiments. SS analyzed the data, with assistance from KB, AYM, SK, and BHP. SS, JV, and CM wrote the manuscript.

## Supplementary Material

Supplemental data

## Version 1 01/13/2022

In-Press Preview

## Version 2 03/01/2022

Electronic publication
