# Supplementary material for: Single-cell analysis of somatic mutation burden in mammary epithelial cells of pathogenic BRCA1/2 mutation carriers
Source: J Clin Invest. 2022 Mar 1;132(5):e148113. doi: 10.1172/JCI148113 (PMC8884908; doi:10.1172/JCI148113)
Supplement: Supplemental data [file jci-132-148113-s165.pdf]

Single-cell analysis of somatic mutation burden in mammary epithelial cells of pathogenic *BRCA1/2* mutation carriers

Supplemental Methods

Supplementary Figure 1. FACS enrichment strategy and gating of human mammary epithelial cells by means of flow cytometry.

Supplementary Figure 2. Distribution of mutation levels in human mammary epithelial cells.

Supplementary Figure 3. Mutational spectra of SNV detected in human mammary epithelial cells.

Supplementary Figure 4. Mutational spectra of INDEL detected in human mammary epithelial cells.

Supplementary Figure 5. Shared mutations in single cells from each individual.

Supplementary Table 1. Demographic and clinical variables of subjects enrolled in the study.

Supplementary Table 2. Whole-genome sequencing data and calls of somatic mutations of human mammary epithelial cells.

Supplementary Table 3. Pathogenic germline variations in bulk cells and somatic mutations in single cells including a subgroup of those mapping to genes with known functions in DNA repair and genome maintenance.

Supplementary Table 4. Somatic mutations of the outlier cell M10-1 mapped to gene regions.

Supplementary Table 5. Correlation of mutational signatures identified in human mammary epithelial cells with cancer-related signatures (COSMIC) and hierarchical Dirichlet process (hdp) extracted signatures.

Supplementary Table 6. Number of shared mutations detected in each subject.

Supplemental Methods

Human specimens

Primary human mammary gland tissue samples were obtained from the Cooperative Human Tissue Network - CHTN (IRB approved protocol #2007-433) (Supplementary Table 1). Seven tissue samples included in the control group were obtained from women of 28-48 years old (mean age 40.0 $SD^{+/-}$7.9) undergoing reduction mammoplasty for cosmetic reasons who reported no previous family history of breast cancer. The high breast cancer risk group included eight patients diagnosed with pathogenic germline mutations in *BRCA1* (n=6) or *BRCA2* (n=2) (mean age 41.1 $SD^{+/-}$2.9); five tissue samples were collected from cancer-free female patients undergoing prophylactic tumor preventive mastectomy (all five patients diagnosed with a germline *BRCA1* pathogenic mutation), three samples were non-tumor mammary tissue samples collected from patients diagnosed with early onset breast cancer (≤42 year old), diagnosed with a pathogenic germline mutation in *BRCA1* (one patient) or *BRCA2* (two patients).

About 1.5-12 grams of mammary gland tissue were delivered within 24 h of surgery in cold RPMI medium at 4°C. Tissue processing and single mammary epithelial cells isolation and collection were performed within 12-16 hours from sample delivery.

Human mammary gland tissue processing

The mammary gland tissue was dissected into smaller pieces of ~3-4 mm using a scalpel and digested as described (1) in 15 ml of digestion solution with 190 U/ml Collagenase III, 120 U/ml hyaluronidase and 100 U/ml DNase (Worthington Biochemical) in DMEM/F-12 Ham (Gibco, Thermo Fisher Scientific) supplemented with 5% FBS, 5µM $CaCl_2$, 2 mM glutamine, 10 µg/ml insulin, 100 U/ml penicillin, 100 µg/ml streptomycin, for 5 to 8 h while gentle shaking at 37˚C. The resulting organoids were washed with PBS by centrifugation for 5 min at 550 g and

sequentially digested with 2 ml of 0.25% trypsin and 1 mM EGTA (3 min, 37˚C) followed by inactivation with 5% FBS in PBS and filtering through a 40 µm cell strainer (BD-Falcon) to obtain single cell suspension. Single cells were washed in PBS by centrifugation for 5 min at 550 g and resuspended in 150 µl of ice-cold PBS for the following labeling with cell specific antibodies.

Single primary mammary epithelial cells collection

Total mammary cell suspensions isolated from primary tissue samples were used to selectively collect single mammary epithelial cells, e.g., basal and luminal cells, into individual 0.2 ml PCR-tubes preloaded with 3 µl of PBS by means of fluorescent activated cell sorting (FACS; FACSAria, Becton Dickenson). To selectively target and collect specific cell populations, total suspensions were incubated with lineage specific and mammary cell specific antibodies as described (2). Mammary cell suspensions in 130 µl PBS were first preincubated with 15 µl of FcR blocking reagent (Miltenyi) for 10 min, followed by incubation with 35 µl of each APC-conjugated antibody to CD49f and PE-Vio770-conjugated antibody to CD326 (EpCAM) (Miltenyi) for 20 min at 4˚C. Cells were washed with 1 ml of PBS by centrifugation for 3 min at 3500 rpm, resuspended in 100 µl of PBS and incubated with 45 µl of each biotinylated Lineage specific antibodies, namely CD31, CD45, CD235a, CD140b (Miltenyi) for 20 min at 4˚C. After subsequent wash, the cells were resuspended in fresh 100 µl of PBS and incubated with 2.5 µl of PE–conjugated streptavidin (Miltenyi) for 10 min at 4˚C. After the final wash Abs-targeted cells were resuspended in 1 ml of PBS with 5 µM EDTA and incubated with 1.5 µl of 5 µM Sytox Green (Invitrogen), 4.5 µl of 10 mg/ml Hoechst 33342 (Invitrogen) and 4.5 µl of 5 mM Reserpine (Sigma) for 20 min at 4˚C. The final suspension was used for selective discrimination and sorting of viable (Sytox Green^{-}), diploid cells (Hoechst 33342^{+} 2n population) carrying basal epithelial cell phenotype Lin^{-} CD49f$^{+/high}$ EpCAM$^{-/low}$ or luminal epithelial cell phenotype Lin^{-} CD49f$^{-/low}$

EpCAM$^{+/high}$. Typical FACS sorting lay-out is shown in **Supplementary Figure 1**. Upon single cell sorting tubes were frozen on dry ice and kept at -80˚C until use.

Human immortalized mammary epithelial cell lines

Noncancerous human mammary epithelial cell line (HMEC) immortalized by introduction and overexpression of catalytic subunit of human telomerase (hTERT-IMEC wildtype; wt) and its genetically modified clones were kindly provided by Dr. Ben Ho Park (Vanderbilt University Medical Center, Nashville, TN). hTERT-IMEC cell line exhibit a mammary tissue basal-like phenotype generally correlated with the origin of *BRCA1* breast tumors (3). Isogenic knock-in cell clones harboring heterozygous mutant *BRCA1* two-bp deletion at the coding region (185delAG) were obtained by gene targeting of the hTERT-IMEC wt cells (*BRCA1* het #1 and #2) as described (3). It has been demonstrated that heterozygous mutant *BRCA1* cell clones with 185delAG could undergo higher degree of gene copy number loss than non-mutant controls. Cells were cultured at 37˚C and 5% CO_2 in MEGM (Clonetics CC-3051) to 80% confluence and split by using a trypsin reagent pack (Clonetics CC-5034).

Single immortalized mammary epithelial cells collection

Single cells from wt and two isogenic clones *BRCA1* het #1 and #2 derived parent clones and their kindred single cells were prepared and collected using automated CellRaft AIR™ System (Cell Microsystems) as described previously with certain adaptation for automated raft isolation (4). Briefly, three hTERT-IMEC cell populations were plated on a three individual sections of CellRaft quad array at the required density of 1000 cells per quad section. After 8-10 hours individual cells were elongated and attached to the array surface on individual rafts. After attachment, the medium with floating cells was replaced, rafts with individual cells were positioned and systematically dislocated from the array with a positioned automatic needle and

transferred with a magnetic wand to 0.2 µl PCR strips containing 2.5 µl PBS. Presence of a single raft was observed under a magnifying light microscope. Upon single-cell collection, strips were fast frozen on dry ice and kept on -80˚C until further use.

Single cell whole genome amplification

Single primary and immortalized mammary epithelial cells were subjected to whole genome amplification (WGA) using the advanced single cell multiple displacement amplification method (SCMDA) as we described (4). As positive and negative controls for WGA human genomic DNA and DNA-free PBS solution were used, respectively. Resultant MDA products were purified using AMPureXP-beads (Beckman Coulter), amplified DNA concentration was measured with Qubit High Sensitivity dsDNA kit (Invitrogen Life Sciences). To control the quality of amplified single cell MDA products, the 8-target locus-dropout tests were performed as described previously (4). Qualified samples (2-8 single cell MDA products per each individual subject and immortalized cell population) were further subjected to library preparation and whole-genome sequencing (WGS).

Genomic DNA extraction

Bulk genomic DNA for respective single cell collections was isolated from small 100-500 µg pieces of mammary gland tissues and total hTERT-IMEC wt cell suspension using DNeasy Blood & Tissue Kit (Qiagen) according to the manufacturer's protocol. DNA concentration was quantified with Qubit High Sensitivity dsDNA kit (Invitrogen Life Sciences) and DNA quality was controlled by 1% agarose gel electrophoresis.

Library preparation and whole genome sequencing

The libraries for Illumina next-generation WGS were generated from 0.4 µg genomic DNA and single cell MDA DNA human samples using NEBNext Ultra II FS DNA Library Prep Kit for

Illumina (New England Biolabs). The libraries were sequenced with 2 x 150 bp paired-end reads on Illumina HiSeq X Ten and NovaSeq sequencing platforms by Novogene, Inc.

Next generation WGS at a minimal depth of 20X base coverage was performed on 2-8 individual cells per each human subject (15 individuals, 64 single cells in total) and each immortalized cell population (3 cell populations, e.g., hTERT-IMEC wt, *BRCA1* het #1 and #2; 8 single cells in total) (Supplementary Table 2), as well as respective bulk DNA (16 bulk DNA samples in total).

Alignment for whole-genome sequencing

The raw sequencing reads were trimmed to remove adapter and low-quality nucleotides by Trim Galore (version 0.3.7). The trimmed reads were aligned to the human reference genome (GRCh37 with decoy) using BWA (mem; version 0.7.13) (5). PCR duplications were removed by samtools (rmdup; version 0.1.19) (6). To correct mapping errors made by genome aligners, the known indels and SNPs were collected from the 1000 Genomes Project (phase I) and dbSNP (build 144). Then indels realignment and base quality score recalibration were performed based on known indels and SNPs via Genome Analysis Toolkit (GATK, version 3.5.0) (7).

Calling germline variants from bulk sequencing

Germline SNVs and small INDELs were called by HaplotypeCaller (7). The variants with GATK quality score ≥ 30 were maintained and further filtered based the recommendation of GATK as 'QD < 2.0, FS > 60.0, MQ < 40.0, MQRankSum < -12.5, ReadPosRankSum < -8.0, SOR > 3.0' for SNV and 'QD < 2.0, FS > 200.0, ReadPosRankSum < -20.0, SOR > 10.0' for INDEL. The clinic pathogenicity of variants were annotated using snpEff (version 4.3t) (8) with annotations from ClinVar (version 20211120) (9). Germline copy number variants (CNVs) were identified by CNVnator (version 0.4.1) (10) with bin size at 100 bp. The outputs of CNVnator with

e-values (e-val1, e-val2, e-val3 and e-val4) ≥ 0.00001, q0 < 0 and q0 ≥ 0.5 were further filtered (11). The calling CNVs were annotated using AnnotSV (version 3.0.5) (12). The pathogenic CNVs were selected when meeting the following criteria: pathogenic in ACMG_class, and completely overlapped with known pathogenic genes or genomic regions. DNA repair gene list was obtained from https://www.mdanderson.org/documents/Labs/Wood-Laboratory/human-dna-repair-genes.html (version June 10th, 2020). The list of genome maintenance genes were obtained from MacRae *et al*. (13).

Calling somatic small variants

Somatic mutations between each single cell and the corresponding bulk were identified by SCcaller (version 2.0.0) (4). Known heterozygous SNPs were called from bulk DNA using HaplotypeCaller (7). To obtain high-quality mutation calls, we only considered heterozygous SNPs with position coverage ≥ 20X, GATK phred-scaled quality score ≥ 30 and dbSNP annotations. The identified somatic mutations whose depth smaller than 20X or supported by bulk data were filtered as default. For INDELs, we further required 30X depth and phred-scaled quality score ≥ 25 to maintain high accuracy results. Mutations overlapping with known SNPs in dbSNP were also annotated and removed using SnpEff (8). As all the samples were from female subjects, we included all mutations on all autosomes and chromosome X for analysis. To exclude cells with high allelic bias, we evaluated the sensitivity of variant caller, which was estimated from ratio of known heterozygous SNPs that were also called in the same cell. All reported mutations were annotated using VEP (version 102) (14), while deleteriousness of mutations was scored by CADD (version 1.6) (15). The phylogenetic tree in hTERT-IMECs was inferred and plotted with PhISCS-BnB (version 1.1) (16).

Estimating mutation frequencies

The frequency of somatic SNVs per cell was estimated after normalizing genomic coverage and calling sensitivity:

$$\text{frequency of somatic mutations per cell} = \frac{\#\ \text{somatic mutations}}{\frac{\text{surveyed genome}}{\text{total size of genome}} * \text{sensitivity}}$$

The surveyed genome per single cell was calculated as the number of nucleotides with read mapping quality ≥ 40 in single cell, read mapping quality ≥ 20 in corresponding bulk data and position coverage ≥ 20X in both single cell and its bulk data. For INDEL, we further required position coverage ≥ 30X in single cell.

Identifying mutation signatures

To identify mutation signature in SNVs, we used non-negative matrix factorization (NMF) and confirmed the results with hierarchical Dirichlet process (hdp). In NMF method, the identified SNVs in all individuals were pooled into five groups: primary cells from control groups and *BRCA1/2* mutant carriers, immortalized cells from wt and *BRCA1* deficient cells, and the outlier primary cell. We revealed three group-specific mutational signatures using the R package "MutationalPatterns" (17). We applied mmsig (18) to fit three mutational signatures to the mutational catalog of each group using expectation maximization algorithm. The 95% confidential intervals of relative contributions for each signature were estimated first by generating 1000 mutational profiles from the multinomial distribution and second by repeating the fitting procedure for each profile (19). In the hdp method, the signatures were extracted from mutations in cell level (https://github.com/nicolaroberts/hdp). Five independent posterior sampling chains were performed separately. Each chain started from 10 random clusters (parameter 'initcc') followed by 5000 burn-in iterations (parameter 'burnin'); 200 iterations were collected (parameter 'n') with a spacing of 200 iterations (parameter 'space'). The cosine similarity was used for comparison of

NMF and hdp methods (Supplementary Figure 3B), indicating that there are three consistent signatures (cosine similarity > 0.95). Because the results are highly similar, we only present the results obtained from the NMF method. For INDELs, we only applied the NMF method and extracted two signatures from the four experimental groups: primary HMECs from control groups and *BRCA1/2* mutant carriers, and hTERT-IMECs from wt and *BRCA1* deficient cells. The hdp method excluded the analysis of INDELs due to their low number detected in most cells.

To identify the potential origin of the mutational spectra, NMF signatures were compared with cancer mutation signatures from COSMIC database (https://cancer.sanger.ac.uk/cosmic/signatures/, version 3.2) (20, 21). The cosine similarity between newly identified and published signatures was calculated in Supplementary Table 5. We also fitted the cancer mutation signatures to our mammary epithelial cells to evaluate the relative contribution of these signatures using MutationalPatterns (fit_to_signatures_strict). Signatures marked as possible sequencing artefacts in COSMIC were not included in the analysis.

The outlier cells

One HMEC cell in the control group, M10-1, was found containing substantially higher SNV burden than any other HMEC cells (Figure 1C, right panel; Supplementary Table 2). The mutation frequency of this cell was higher than Q3 + 3 * IQR, where Q3 is the third quartile of the frequencies and IQR is the inter-quartile range. The surveyed genome and the sensitivity of this outlier cell are both the eighth lowest of what observed among all primary HMECs (Supplementary Figure 2B). Additionally, in M10-1, we found high contribution of signature M1 which is the specific signature enriched in the *BRCA1/2* mutant group (Figure 2B). Thus, we excluded this cell from all statistical evaluation in the group comparisons based on SNVs and separated it as a single group in SNV signature analysis. M10-1 did not display high frequency of INDELs, which were

observed in two other cells with high frequencies (Supplementary Table 2). As no further evidence for technical bias was found in these two cells, they were retained in their original groups during the comparison analysis.

Statistical analyses

To compare the levels of mutation frequency in hTERT-IMEC wt and *BRCA1* mutant cells and the median mutations per sample in the primary HMECs experimental groups the negative binomial generalized linear model was applied. For comparisons of mutation frequency levels in single HMECs in the control and in the *BRCA1/2* mutant carrier groups, the negative binomial generalized linear mixed-effect model was used. The differences in SNV signatures between HMECs control and *BRCA1/2* mutant carrier were tested using Pearson's Chi-squared test. A P value less than 0.05 was considered significant. The cell M01-4 with SNV sensitivity smaller than 25% was filtered in analysis.

References for supplementary methods

1. Labarge MA, *et al*. Processing of human reduction mammoplasty and mastectomy tissues for cell culture. *J Vis Exp*. Jan 3 2013;(71)doi:10.3791/50011

2. Inman JL, *et al*. Mammary gland development: cell fate specification, stem cells and the microenvironment. *Development*. Mar 15 2015;142(6):1028-42. doi:10.1242/dev.087643

3. Konishi H, *et al*. Mutation of a single allele of the cancer susceptibility gene BRCA1 leads to genomic instability in human breast epithelial cells. *Proc Natl Acad Sci U S A*. Oct 25 2011;108(43):17773-8. doi:10.1073/pnas.1110969108

4. Dong X, *et al*. Accurate identification of single-nucleotide variants in whole-genome-amplified single cells. *Nat Methods*. May 2017;14(5):491-493. doi:10.1038/nmeth.4227

5. Li H, Durbin R. Fast and accurate short read alignment with Burrows-Wheeler transform. *Bioinformatics*. Jul 15 2009;25(14):1754-60. doi:10.1093/bioinformatics/btp324

6. Li H, *et al*. The Sequence Alignment/Map format and SAMtools. *Bioinformatics*. Aug 15 2009;25(16):2078-9. doi:10.1093/bioinformatics/btp352

7. McKenna A, *et al*. The Genome Analysis Toolkit: a MapReduce framework for analyzing next-generation DNA sequencing data. *Genome Res*. Sep 2010;20(9):1297-303. doi:10.1101/gr.107524.110

8. Cingolani P, *et al*. A program for annotating and predicting the effects of single nucleotide polymorphisms, SnpEff: SNPs in the genome of Drosophila melanogaster strain w1118; iso-2; iso-3. *Fly (Austin)*. Apr-Jun 2012;6(2):80-92. doi:10.4161/fly.19695

9. Landrum MJ, *et al*. ClinVar: improvements to accessing data. *Nucleic Acids Res*. Jan 8 2020;48(D1):D835-D844. doi:10.1093/nar/gkz972

10. Abyzov A, *et al*. CNVnator: an approach to discover, genotype, and characterize typical and atypical CNVs from family and population genome sequencing. *Genome Res*. Jun 2011;21(6):974-84. doi:10.1101/gr.114876.110

11. Jeon S, *et al*. Korean Genome Project: 1094 Korean personal genomes with clinical information. *Sci Adv*. May 2020;6(22):eaaz7835. doi:10.1126/sciadv.aaz7835

12. Geoffroy V, *et al*. AnnotSV: an integrated tool for structural variations annotation. *Bioinformatics*. Oct 15 2018;34(20):3572-3574. doi:10.1093/bioinformatics/bty304

13. MacRae SL, *et al*. Comparative analysis of genome maintenance genes in naked mole rat, mouse, and human. *Aging Cell*. Apr 2015;14(2):288-91. doi:10.1111/acel.12314

14. McLaren W, *et al*. The Ensembl Variant Effect Predictor. *Genome Biol*. Jun 6 2016;17(1):122. doi:10.1186/s13059-016-0974-4

15. Rentzsch P, *et al*. CADD-Splice-improving genome-wide variant effect prediction using deep learning-derived splice scores. *Genome Med*. Feb 22 2021;13(1):31. doi:10.1186/s13073-021-00835-9

16. Sadeqi Azer E, *et al*. PhISCS-BnB: a fast branch and bound algorithm for the perfect tumor phylogeny reconstruction problem. *Bioinformatics*. Jul 1 2020;36(Suppl_1):i169-i176. doi:10.1093/bioinformatics/btaa464

17. Blokzijl F, *et al*. MutationalPatterns: comprehensive genome-wide analysis of mutational processes. *Genome Med*. Apr 25 2018;10(1):33. doi:10.1186/s13073-018-0539-0

18. Rustad EH, *et al*. mmsig: a fitting approach to accurately identify somatic mutational signatures in hematological malignancies. *Commun Biol*. Mar 29 2021;4(1):424. doi:10.1038/s42003-021-01938-0

19. Rustad EH, *et al*. Timing the initiation of multiple myeloma. *Nat Commun*. Apr 21 2020;11(1):1917. doi:10.1038/s41467-020-15740-9

20. Alexandrov LB, *et al*. The repertoire of mutational signatures in human cancer. *Nature*. Feb 2020;578(7793):94-101. doi:10.1038/s41586-020-1943-3

21. Tate JG, *et al*. COSMIC: the Catalogue Of Somatic Mutations In Cancer. *Nucleic Acids Res*. Jan 8 2019;47(D1):D941-D947. doi:10.1093/nar/gky1015

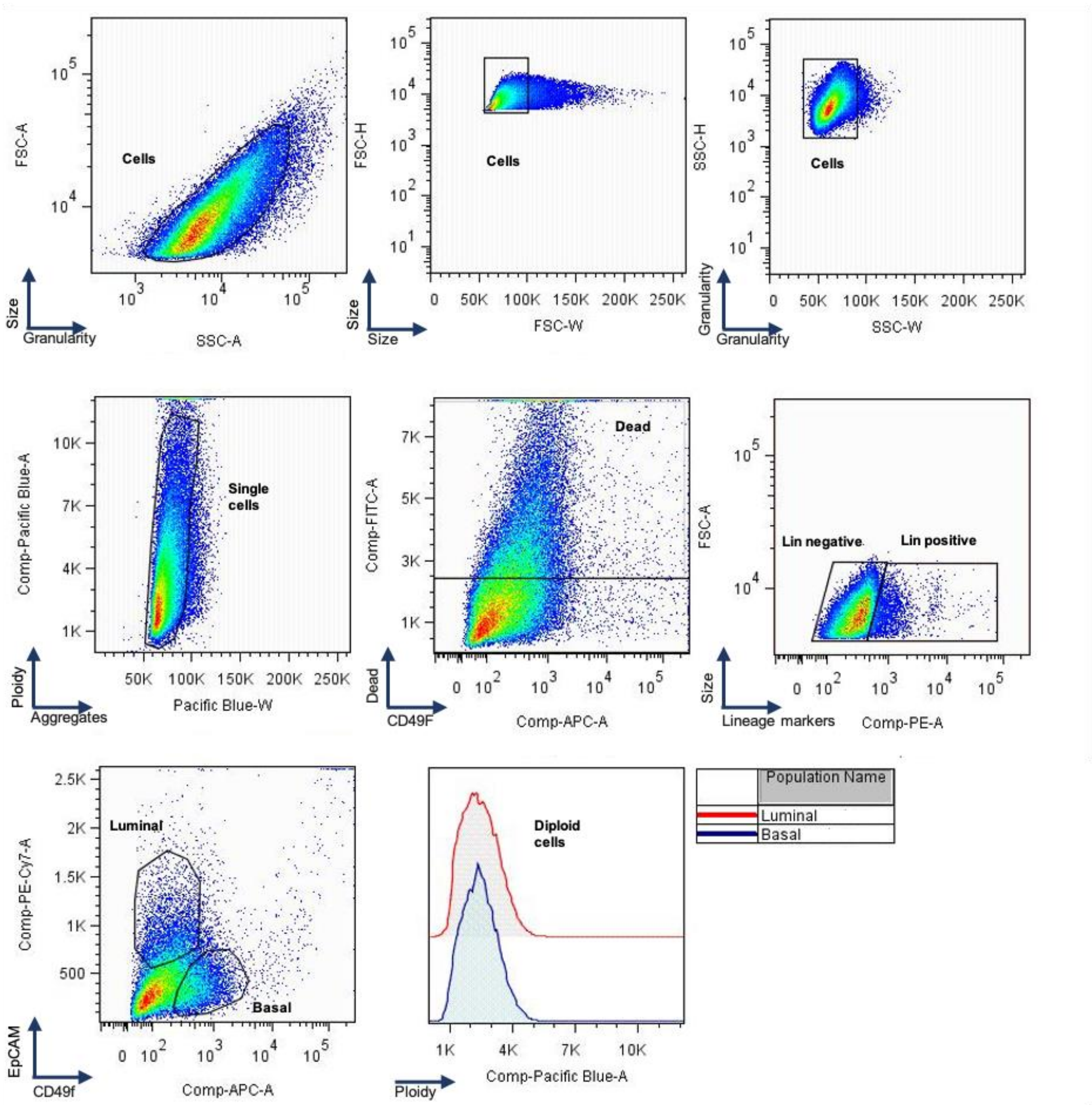

Supplementary Figure 1. FACS enrichment strategy and gating of human mammary epithelial cells by means of flow cytometry. The first row displays the gating of cells based on size and granularity; the second row displays the gating for the removal of dead cells and the lineage negative selection; the bottom row displays the gating of mammary epithelial luminal or basal cells based on the expression of lineage specific markers described in the methods section.

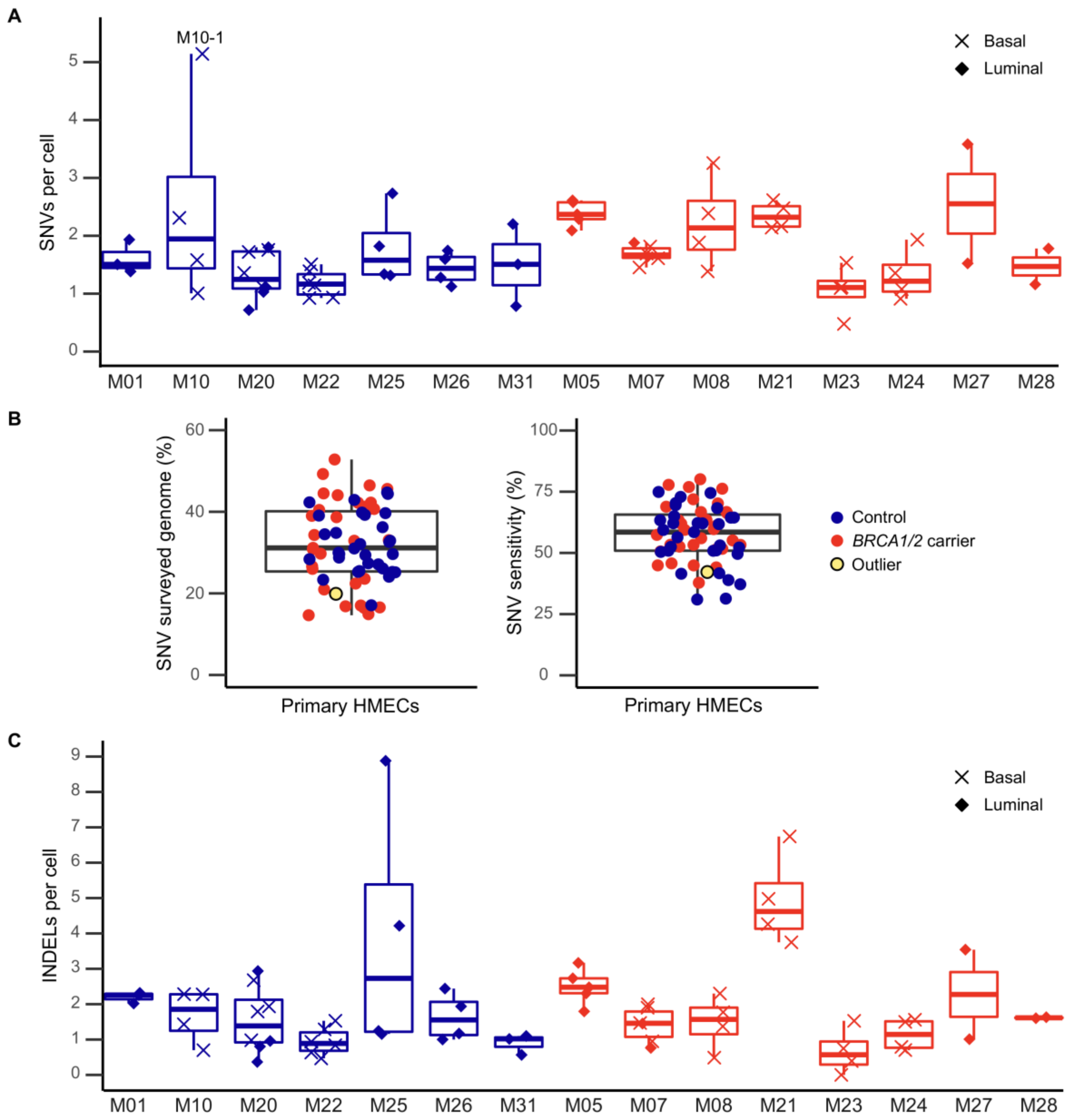

Supplementary Figure 2. Distribution of mutation levels in human mammary epithelial cells. (**A**) Numbers of SNV in single HMECs measured in controls (blue) and *BRCA1/2* mutant carriers (red). (**B**) The distribution of the surveyed genome (left panel) and the sensitivity (right panel) in SNV detection are depicted. Each dot corresponds to one cell. The outlier cell M10-1 in the control group is depicted in yellow and it is the eighth lowest among levels observed across all the cells in both analyses. (**C**) Numbers of INDEL in single HMECs cells measured in controls (blue) and *BRCA1/2* mutant carriers (red).

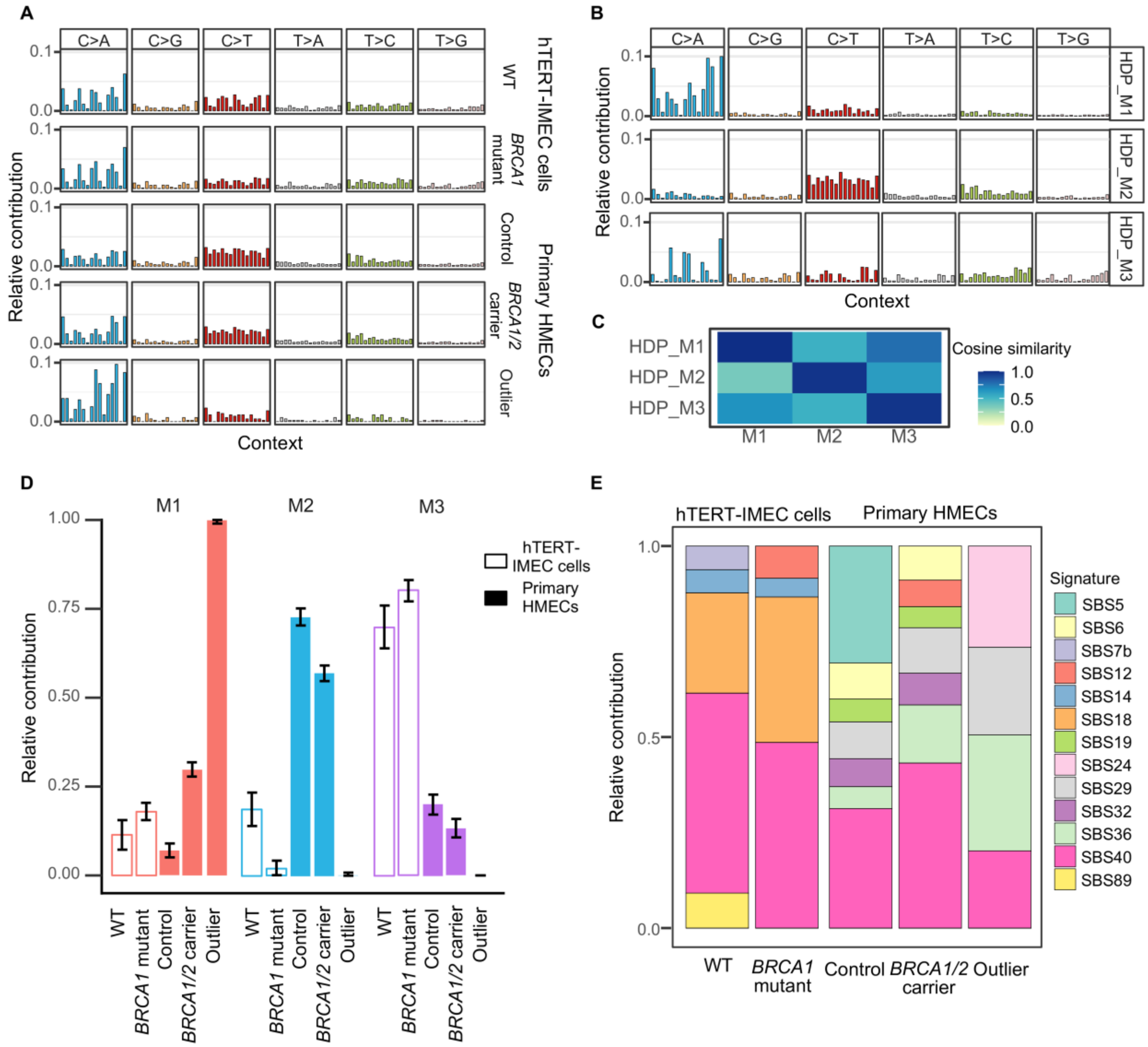

Supplementary Figure 3. Mutational spectra of SNV detected in human mammary epithelial cells.

(**A**) Bar graph depicting the 96-mutational type spectra of the five groups of human mammary epithelial cells. (**B**) Three de novo mutational signatures were identified by hierarchical Dirichlet process (hdp) from the somatic mutations of all single cells. (**C**) Heatmap of cosine similarity between signatures extracted by non-negative matrix factorization (NMF) and hdp methods. (**D**) 95% confidence intervals of relative contributions for three NMF signatures depicted in Figure 2B. Data is represented as the mean with 95% confidence intervals. (**E**) The contribution of the COSMIC signatures across the five experimental groups shown in panel (A).

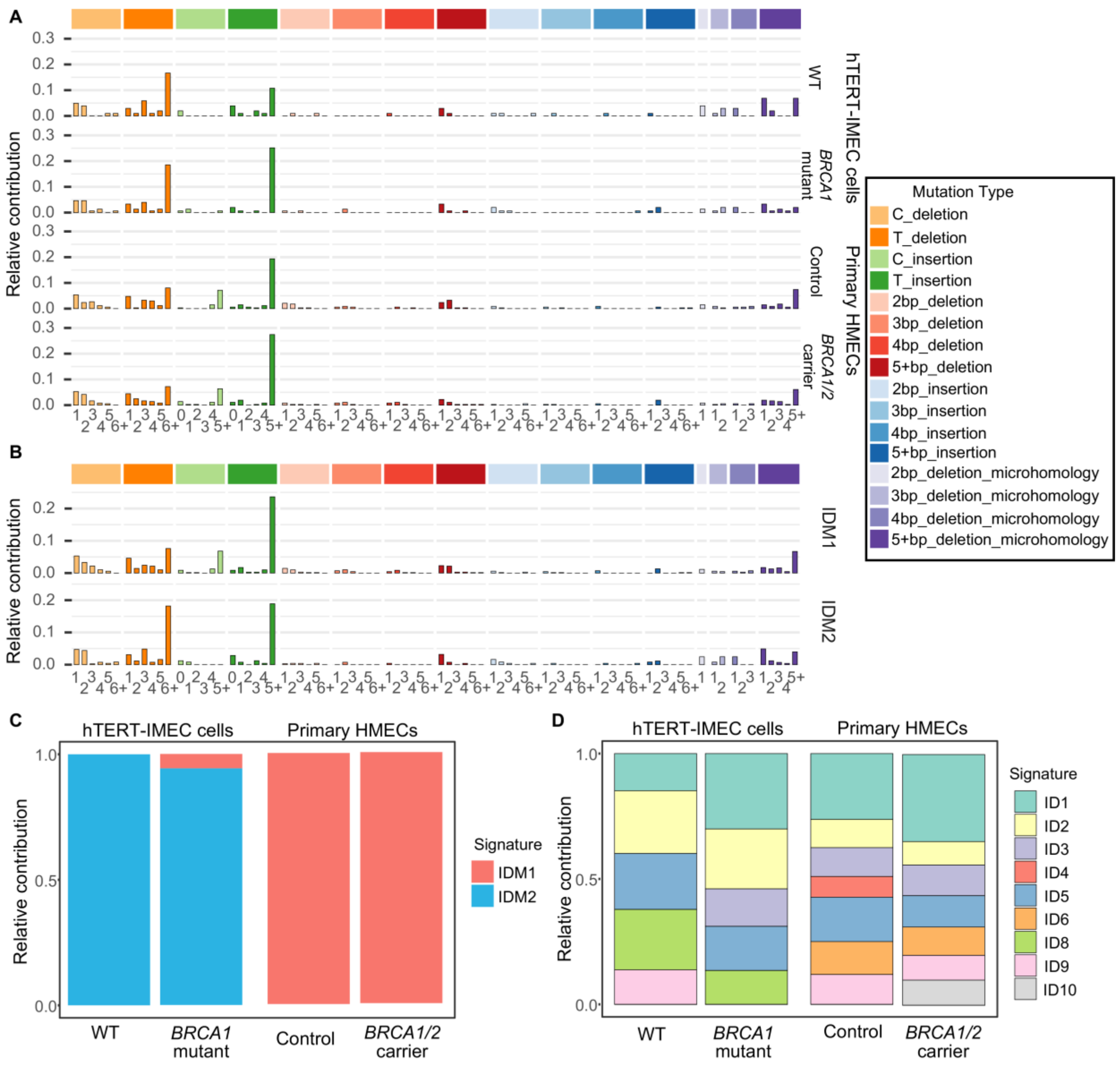

Supplementary Figure 4. Mutational spectra of INDEL detected in human mammary epithelial cells.

(**A**) Bar graph depicting the 83-mutational type spectra detected in four groups of human mammary epithelial cells. The contributions of different types of INDELs were grouped based on the length of INDELs, the affected nucleotides (C or T) and the number of repetitive elements within the repetitive or microhomology region when the INDEL occurred in such a region. (**B**) Two mutational signatures were identified de novo by the NMF method. (**C**) The contributions of signatures IDM1 (blue) and IDM2 (salmon) to all INDELs in these four groups using NMF methods. The bar graphs on the left depict the spectra detected in hTERT-IMEC cells and the bar graphs on the right depict those of the primary HMECs. (**D**) Plot of the contributions of the COSMIC INDEL signatures across the four groups shown in panel (A).

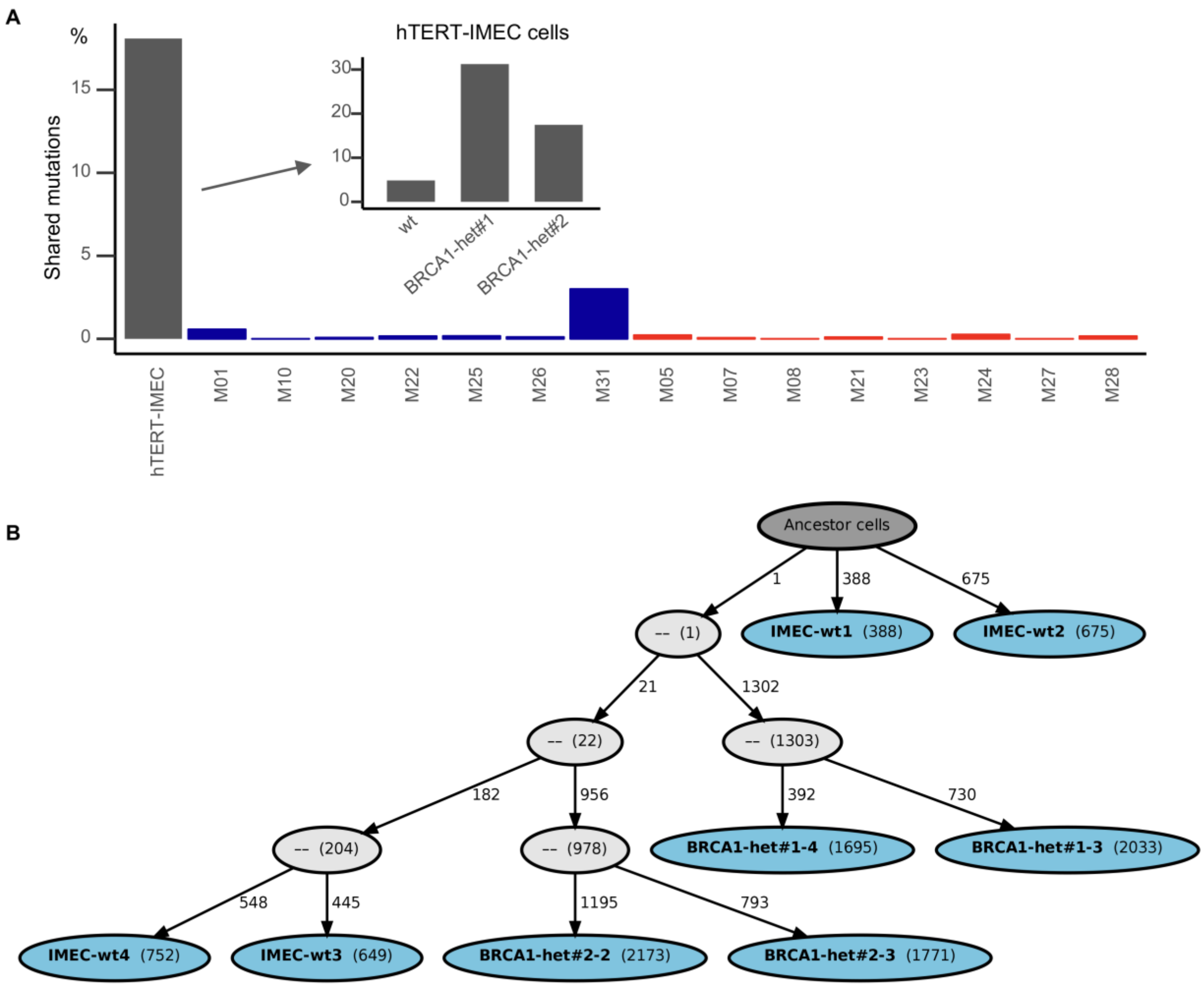

Supplementary Figure 5. Shared mutations in single cells of each individuals.
(**A**) Plot depicting the percentage of shared mutations in each individual. hTERT-IMECs are shown in grey, primary control cells are shown in blue and *BRCA1/2* mutant carriers are shown in red. (**B**) Phylogenetic tree of single cells in hTERT-IMECs. The numbers inside the parentheses indicate the total number of SNVs occurring from the ancestor cell (root) to that node. The numbers on each edge are the SNVs occurring between the associated parent and child nodes. The nodes in blue are the actual single cells.
